# It takes two to tango: NAD^+^ and sirtuins in aging/longevity control

**DOI:** 10.1038/npjamd.2016.17

**Published:** 2016-08-18

**Authors:** Shin-ichiro Imai, Leonard Guarente

**Affiliations:** 1 Department of Developmental Biology, Washington University School of Medicine, St Louis, MO, USA; 2 Department of Biology and Glenn Laboratories for the Science of Aging, Massachusetts Institute of Technology, Cambridge, MA, USA; 3 Koch Institute for Integrative Cancer Research, Massachusetts Institute of Technology, Cambridge, MA, USA

## Abstract

The coupling of nicotinamide adenine dinucleotide (NAD^+^) breakdown and protein deacylation is a unique feature of the family of proteins called ‘sirtuins.’ This intimate connection between NAD^+^ and sirtuins has an ancient origin and provides a mechanistic foundation that translates the regulation of energy metabolism into aging and longevity control in diverse organisms. Although the field of sirtuin research went through intensive controversies, an increasing number of recent studies have put those controversies to rest and fully established the significance of sirtuins as an evolutionarily conserved aging/longevity regulator. The tight connection between NAD^+^ and sirtuins is regulated at several different levels, adding further complexity to their coordination in metabolic and aging/longevity control. Interestingly, it has been demonstrated that NAD^+^ availability decreases over age, reducing sirtuin activities and affecting the communication between the nucleus and mitochondria at a cellular level and also between the hypothalamus and adipose tissue at a systemic level. These dynamic cellular and systemic processes likely contribute to the development of age-associated functional decline and the pathogenesis of diseases of aging. To mitigate these age-associated problems, supplementation of key NAD^+^ intermediates is currently drawing significant attention. In this review article, we will summarize these important aspects of the intimate connection between NAD^+^ and sirtuins in aging/longevity control.

## It’s so long ago

Since the first discovery of nicotinamide adenine dinucleotide (NAD^+^)-dependent deacetylase activity of the silent information regulator 2 (Sir2) family (‘sirtuins’),^1^ the field of sirtuin biology has been evolving rapidly over the past 16 years. Many researchers from different fields have encountered sirtuins in their own research, enriching our knowledge of this fascinating family of enzymes. It is now clear that sirtuins are involved in the regulation of many fundamental biological processes throughout the body.^2,3^ Furthermore, it has been revealed that sirtuins possess much broader enzymatic activities, namely, deacylases, including deacetylase, desuccinylase, demaloynylase, deglutarylase, long-chain deacylase, lipoamidase, and ADP-ribosyltransferase.^4,5^ All these enzymatic activities specifically require NAD^+^, and the catalytic mechanism of this NAD^+^ dependency has been studied extensively.^6^ Clearly, sirtuins have evolved to respond to the availability of NAD^+^, an essential currency of cellular metabolism and DNA damage repair, and convert this information to many different biological outputs. In this particular review article, we will focus on this intimate connection between sirtuin function, aging/longevity control in particular, and their indispensable co-substrate, NAD^+^.

**Figure 1:**
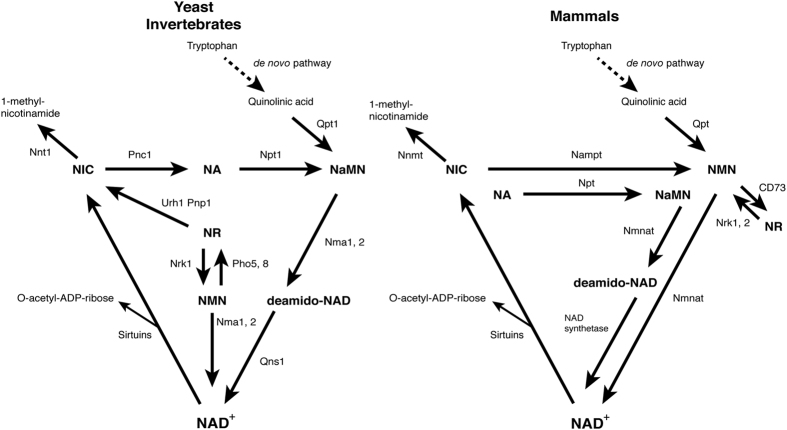
Comparison of NAD^+^ biosynthetic pathways between yeast/invertebrates and mammals. *Left panel*: NAD^+^ biosynthetic pathways in the budding yeast *Saccharomyces cerevisiae* and invertebrates. The pathways from NIC, NA, and NR and the *de novo* pathway from tryptophan are shown. *Right panel*: NAD^+^ biosynthetic pathways in mammals. In mammals, NIC is a predominant NAD^+^ precursor. NA, NR, and tryptophan can also be utilized to synthesize NAD^+^. The *de novo* pathway and the NAD^+^ biosynthetic pathway from NA are evolutionarily conserved, whereas the NAD^+^ biosynthetic pathway from NIC is mediated by NAMPT. In this figure, only sirtuins are shown among multiple NAD^+^-consuming enzymes that break NAD^+^ into nicotinamide and ADP-ribose. Mammals have two NR kinases (Nrk1 and 2) and ecto-5′-nucleotidase CD73 to produce NMN and NR, respectively. NA, nicotinic acid; NAD+, nicotinamide adenine dinucleotide; NAMPT, nicotinamide phosphoribosyltransferase; NIC, nicotinamide; NR, nicotinamide riboside; Nrk1, nicotinamide ribose kinase 1; Npt1, nicotinic acid phosphoribosyltransferase; Nma1, 2, nicotinic acid mononucleotide adenylyltransferase 1, 2; Nnt1, nicotinamide-N-methyltransferase; Npt, nicotinic acid phosphoribosyltransferase; NMNAT, NMN adenylyltransferase; NNMT, nicotinamide-N-methyltransferase; NaMN, nicotinic acid mononucleotide; NMN, nicotinamide mononucleotide; Pho5, 8, phosphatase 5, 8; Pnp1, purine nucleoside phosphorylase; Pnc1, nicotinamidase; Qpt, quinolinic acid phosphoribosyltransferase; Qns1, NAD synthetase; Qpt1, quinolinic acid phosphoribosyltransfease; Urh1, uridine hydrolase.

The origin of the connection between NAD^+^ and sirtuins is ancient. For instance, vibriophage KVP40 possesses a minimal set of genes for NAD^+^ biosynthesis and consumption, namely, the genes encoding two key NAD^+^ biosynthetic enzymes, nicotinamide phosphoribosyltransferase (NAMPT) and nicotinamide/nicotinic acid mononucleotide adenylyltransferase (NMNAT),^2,3^ and a sirtuin family protein (Figure 1).^7^ Although why such a minimalistic organism keeps these three genes in its genome remains unclear, one potential explanation is that controlling the host cell’s metabolism and proliferation in a NAD^+^-dependent manner could provide benefits for this particular vibriophage to efficiently produce progeny in the host cell. Such a NAD^+^/sirtuin-mediated virus–host relationship might be a prototype for the much more complex inter-tissue communication mediated by NAMPT and the mammalian sirtuin SIRT1.^8^ As discussed later in this review, NAMPT and SIRT1 comprise multiple layers of feedback regulatory loops inside cells and between tissues and organs, and contribute to the systemic regulation of mammalian aging and longevity.^9–12^ Another interesting example is the connection between the nicotinamidase Pnc1 and Sir2 proteins in yeast, worms, and flies. Whereas vertebrates and a limited number of bacterial species mainly use NAMPT to synthesize NAD^+^ from nicotinamide, invertebrates and most bacterial species use nicotinamidase to convert nicotinamide to nicotinic acid and synthesize NAD^+^ from nicotinic acid (Figure 1).^13^ Pnc1 regulates NAD^+^ biosynthesis and affects lifespan in those organisms.^14–16^

Although all genetic, pathophysiological, and pharmacological studies point out that sirtuin activity can be affected by changes in NAD^+^ levels, whether sirtuin activity is indeed regulated by the physiological fluctuation of NAD^+^ has been of great debate. However, a recent detailed kinetic study has demonstrated that at least for deacetylase activities of SIRT1-3, each *K*_m_ for NAD^+^ is consistent with the notion that changes in NAD^+^ levels in each subcellular compartment can directly regulate sirtuin activity.^17^ These findings further affirm the functional connection between NAD^+^ and sirtuins. We will now look into several important cases of this connection in diverse organisms.

## Learned all the games

The history of sirtuin research in the past 16 years has been a steep winding road. In this history, the biggest controversy was whether sirtuins are truly an evolutionarily conserved regulator for aging and longevity in diverse organisms. Although early studies demonstrated that Sir2 and its orthologs have a critical role in aging/longevity control in yeast, worms, and flies,^18–20^ subsequent studies reported contradictory results,^21,22^ bringing considerable debates on the role of sirtuins in aging/longevity control. However, this controversy has finally been put to the end because an increasing number of recent studies have successfully reconfirmed the original claims.^23–30^ Even in mammals, the brain-specific SIRT1-overexpressing (BRASTO) transgenic mice have been demonstrated to exhibit significant delay in aging and lifespan extension in both male and female mice.^27^ Given that the whole-body SIRT1-overexpressing transgenic mice fail to show lifespan extension,^22^ this study strongly suggests that SIRT1 in the brain, more specifically in the hypothalamus, is a key to control aging and longevity in mammals.^31^ In addition, whole-body SIRT6 transgenic mice have been reported to show lifespan extension, although only males exhibit the phenotype.^24^ These recent studies have established a firm foundation for the significance of sirtuins as an evolutionarily conserved aging/longevity regulator.^32–34^

**Figure 2:**
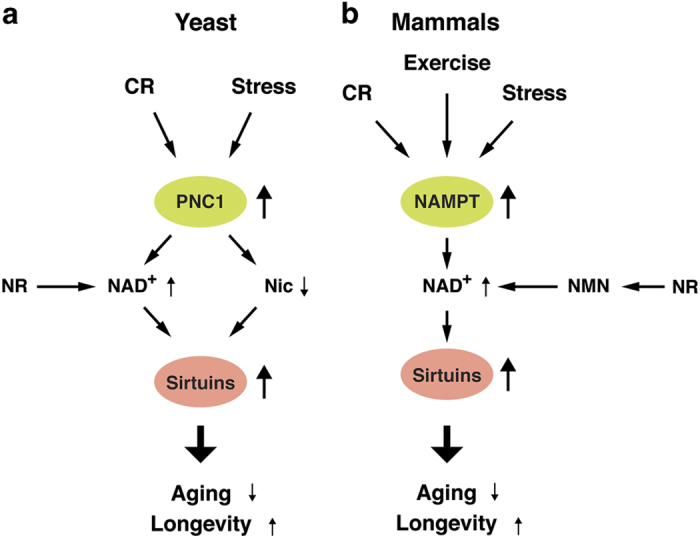
Responses of NAD^+^ biosynthetic enzymes and sirtuins to nutritional and environmental cues in aging/longevity control. (**a**) Responses of PNC1 and sirtuins in budding yeast *Saccharomyces cerevisiae* and invertebrates (worms and flies) to caloric restriction (CR) and stress. When PNC1 levels increase in response to CR or stress in these organisms, the NAD^+^ biosynthetic flux increases and nicotinamide levels decrease, both contributing to the enhancement of sirtuin activity. (**b**) Responses of NAMPT and sirtuins in mammals to CR, exercise, and stress. Upregulation of NAMPT activates sirtuins primarily through the increase in NAD^+^ biosynthesis because nicotinamide levels are normally much lower in mammals, compared to yeast. CR, caloric restriction; NAD^+^, nicotinamide adenine dinucleotide; NAMPT, nicotinamide phosphoribosyltransferase.

Interestingly, the connection between NAD^+^ and sirtuins has also been demonstrated to be crucial in aging/longevity control (Figure 2). In yeast, caloric restriction (CR), achieved by lowering glucose in the media from regular 2% down to 0.5% or 0.05%, substantially extends lifespan, and the lifespan extension by moderate CR (0.5% glucose) is dependent on Sir2 or nicotinic acid phosphoribosyltransferase Npt1.^35^ A more severe regimen of CR (0.05% glucose) extends lifespan by a different mechanism that is independent of Sir2 but dependent of the nutrient-responsive kinases, PKA, TOR, and the Akt ortholog Sch9.^36^ In addition, induction of nicotinamidase Pnc1, which is upstream of Npt1, is necessary and sufficient for lifespan extension by CR (Figure 2a).^14^ Nicotinamide riboside (NR), a key NAD^+^ intermediate, is produced and secreted by yeast cells,^37,38^ and exogenous NR increases NAD^+^ biosynthesis and promotes lifespan through two independent pathways, the one mediated by NR kinase Nrk1 and the other mediated by uridine hydrolase Urh1 and purine nucleoside phosphorylase Pnp1.^39^ In worms, lifespan extension mediated by CR requires Sir-2.1 and Pnc1.^40^ In addition, NR is also able to promote lifespan in a Sir-2.1-dependent manner.^25^ Overexpression of the *Drosophila *nicotinamidase, D-NAAM, also extends lifespan in flies, and this lifespan extension is Sir2-dependent.^15^ These findings strongly suggest the importance of the connection between NAD^+^ and sirtuins in aging/longevity control in these organisms, which utilize nicotinic acid and nicotinamide ribose as main sources for NAD^+^ biosynthesis.

**Figure 3:**
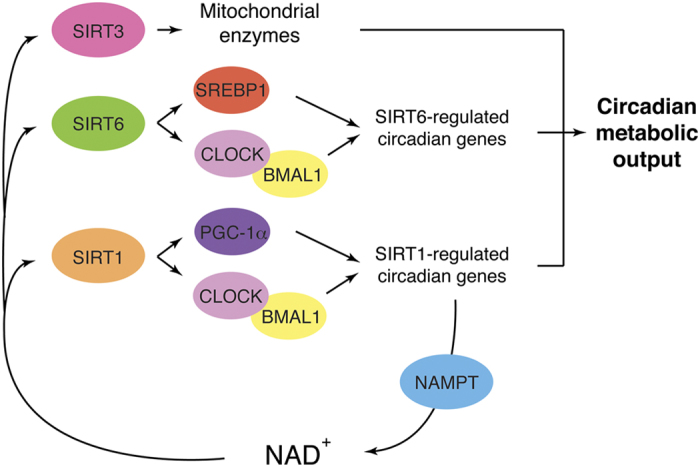
Circadian regulation of NAD^+^ biosynthesis and metabolism by NAMPT and sirtuins. *Nampt* is one of the SIRT1/CLOCK/BMAL1-regulated circadian genes, and SIRT1 and NAMPT comprise a novel circadian regulatory feedback loop, producing the circadian oscillation of NAD^+^. This circadian oscillation of NAD^+^ drives SIRT1, SIRT3, and SIRT6 activities. SIRT1 feedbacks the key circadian transcription factors CLOCK/BMAL and regulates genes related to peptide and cofactor biosynthesis in the liver. SIRT1 also regulates *Bmal1* expression through PGC-1α in the suprachiasmatic nucleus. SIRT6 controls the chromatin recruitment of CLOCK/BMAL1 and SREBP1 and regulates genes related to lipid and carbohydrate metabolism. SIRT3 regulates oxidative metabolism in mitochondria through circadian deacetylation of mitochondrial oxidative enzymes. All these circadian activity changes of sirtuins produce robust metabolic outputs in many different tissues and organs. NAD^+^, nicotinamide adenine dinucleotide; NAMPT, nicotinamide phosphoribosyltransferase.

Different from yeast, worms, and flies, mammals do not have Pnc1 homologs but utilize NAMPT instead, converting nicotinamide, an amide form of vitamin B_3_, and 5′-phosphoribosyl-pyrophosphate to nicotinamide mononucleotide (NMN), another key NAD^+^ intermediate (Figure 1).^41,42^ NMN is adenylated to NAD^+^ by three NMN adenylyltransferases, NMNAT1-3. It has been well established that the NAMPT-mediated NAD^+^ biosynthetic pathway regulates sirtuin activity in the nucleus and mitochondria.^43–47^ Just like Pnc1 in lower eukaryotes, NAMPT is also induced in rodents and humans by low glucose, fasting, CR, exercise, and a variety of stress and damage (Figure 2b).^47–51^ An unique feature of this particular NAD^+^ biosynthetic pathway is that transcription of the *Nampt* gene is mediated by the key circadian transcription factors CLOCK/BMAL, rendering NAD^+^ biosynthesis and the activity of sirtuins circadian. In addition, SIRT1 and SIRT6 feedback on the circadian oscillator in peripheral tissues and likely in the suprachiasmatic nucleus of the hypothalamus.^43,45,52^ The circadian oscillation of NAD^+^ drives at least SIRT1, SIRT3, and SIRT6 activities, impacting a variety of metabolic functions, including glucose, cholesterol, and fatty acid metabolism (Figure 3).^44,53^

Most recently, another interesting connection between NAMPT/NAD^+^ and SIRT1 has been found.^8^ Mammals have two different forms of NAMPT, intra- and extracellular NAMPT (iNAMPT and eNAMPT, respectively). iNAMPT is acetylated in white and brown adipose tissue. When iNAMPT is specifically deacetylated at lysine 53 by SIRT1, the protein is predisposed to secretion, and its enzymatic activity is enhanced. Surprisingly, eNAMPT secreted by adipose tissue remotely controls NAD^+^ biosynthesis, SIRT1 activity, and neural activity in the hypothalamus, which affects physical activity of mice during the dark phase and in response to fasting. These findings imply that adipose tissue communicates with other tissues through the secretion of eNAMPT, and regulates the spatial and temporal coordination of NAD^+^ biosynthesis at a systemic level. Given that the hypothalamus functions as a high-order ‘control center of aging’ in mammals,^27,54^ it is conceivable that adipose tissue functions as a ‘modulator’ of this control center (see Figure 5). Indeed, knock-in mice in which iNAMPT is overexpressed specifically in adipose tissue (ANKI mice) show significant increases in circulating eNAMPT levels, hypothalamic NAD^+^ and SIRT1 target gene expression, and physical activity, particularly in response to fasting.^8^ Therefore, it will be of great interest to examine whether these ANKI mice show a significant slowing in aging and lifespan extension.

Given that NAMPT and NAD^+^ levels display robust circadian oscillation in peripheral tissues,^45^ it is conceivable that eNAMPT secretion would also show circadian oscillation. Indeed, it has been reported that serum eNAMPT levels follow a diurnal rhythm, making a peak during early afternoon, in humans,^55^ which appears to be opposite to plasma eNAMPT oscillation in mice (a preliminary observation in the Imai lab). This eNAMPT oscillation would likely produce another oscillation of its enzymatic reaction product, NMN, in blood circulation. NMN is rapidly incorporated to major metabolic tissues and converted to NAD^+^.^56^ Therefore, in mammals, adipose tissue has an important role in generating the circadian oscillation of eNAMPT and possibly NMN in blood circulation, potentially synchronizing metabolic and neurobehavioral functions through other peripheral tissues and the hypothalamus in a circadian rhythm-dependent manner. In each tissue, NAD^+^ and sirtuins are critical mediators to orchestrate such inter-tissue communications. The loss of this orchestration is likely an important driver of aging in a wide variety of organisms.

## All the things that were said

**Figure 4:**
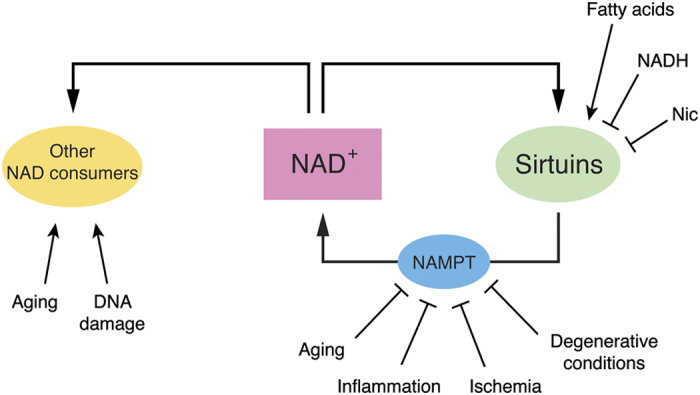
Regulation of the functional connection between NAD^+^ and sirtuins. There are at least three levels of regulation: (1) regulation of NAD^+^ biosynthesis, particularly mediated by NAMPT, (2) modulation of sirtuin activity, including inhibition by nicotinamide and NADH and activation of SIRT6 by long-chain fatty acids, and (3) competition with other NAD^+^-consuming enzymes, such as PARPs and CD38/157 ectoenzymes. See details in text. NAD^+^, nicotinamide adenine dinucleotide; NAMPT, nicotinamide phosphoribosyltransferase; PARPs, poly-ADP-ribose polymerases.

The functional connection between NAD^+^ and sirtuins is regulated at at least three levels: (1) regulation of NAD^+^ biosynthesis, (2) modulation of sirtuin activity by NAD^+^ substrates and derivatives, and (3) competitive utilization of NAD^+^ between sirtuins and other NAD^+^ consumers (Figure 4).

First, the pathophysiological changes in NAD^+^ biosynthesis affect sirtuin activity. For instance, in mammals, NAMPT and NAD^+^ levels decline with age in multiple organs, such as pancreas, adipose tissue, skeletal muscle, liver, and brain.^25,56–58^ Inflammatory, ischemic, and degenerative disease conditions also decrease NAMPT-mediated NAD^+^ biosynthesis.^56,59–62^ Although precise molecular mechanisms for the decline in NAMPT-mediated NAD^+^ biosynthesis remain unclear, it has been suggested that oxidative stress and/or inflammatory cytokines decrease NAMPT expression.^56^ Such pathophysiological decline in NAD^+^ biosynthesis decreases sirtuin activity, likely contributing to the development of age-associated pathophysiologies. For this reason, boosting NAD^+^ biosynthesis by using key NAD^+^ intermediates, such as NMN and NR, is now drawing significant attention as an efficient therapeutic intervention against diseases of aging, such as type 2 diabetes, Alzheimer’s disease, heart failure, and hearing loss.^2,3^ Thus, the regulation of NAD^+^ biosynthesis is one of the most important factors that affect the functional connection between NAD^+^ and sirtuins.

Second, sirtuin activity can also be modulated by NAD^+^ substrates and derivatives, such as nicotinamide and NADH, and other molecules. Nicotinamide, which is released from NAD^+^ during the deacylation reaction of sirtuins, functions as a non-competitive inhibitor.^63^ The ability of nicotinamide to inhibit deacylation by sirtuins is dependent on the type of acyl substrates.^17^ For instance, for SIRT1, the IC_50_ for nicotinamide is 175 μM for the acetylated substrate but significantly less for longer chain acyl substrates. On the other hand, SIRT3 shows an opposite trend for nicotinamide inhibition. Whereas the calculated concentration of endogenous nicotinamide ranges from 10 to 150 μM in yeast,^63^ plasma nicotinamide concentrations range from 0.3 (human) to 5 μM (mouse) in mammals.^64–66^ Therefore, nicotinamide might be more critical for the regulation of sirtuin activity in lower eukaryotes that tend to have higher endogenous nicotinamide concentrations. The NAD^+^/NADH ratio has also been proposed to be a critical parameter for the regulation of sirtuin activity due to the capability of NADH as a competitive inhibitor.^67^ However, the subsequent studies have shown that the IC_50_ for NADH surpasses reported physiological ranges of NADH so that it is unlikely that NADH could function as a physiologically relevant competitive inhibitor for sirtuins, at least in mammals.^44,68,69^ Most recently, it has been demonstrated that the enzymatic activities of some sirtuins, particularly SIRT6, are regulated by long-chain fatty acids.^70^ Several free fatty acids, including myristic, oleic, and linoleic acids, at physiological concentrations are able to enhance SIRT6 deacetylase activity at K9 and K56 of histone H3 up to 35-fold. Taken together, these studies suggest that the connection between NAD^+^ and sirtuins can be modified by physiologically relevant endogenous NAD^+^ derivatives and other compounds.

Lastly, the availability of NAD^+^ is under a fierce competition among NAD^+^-consuming enzymes, including sirtuins, poly-ADP-ribose polymerases (PARPs), and CD38/157 ectoenzymes.^2,3^ Particularly, SIRT1 and PARP1 compete with each other, and genetic ablation and pharmacological inhibition of PARP1 increase NAD^+^ content and SIRT1 activity and enhance oxidative metabolism.^71^ Similar findings were also reported in CD38 knockout mice.^72,73^ Interestingly, it has been shown that PARP is chronically activated in aging worms and mice, resulting in an increase in poly-ADP-ribosylation of cellular proteins.^25^ This chronic PARP activation, which might potentially be caused by an increase in chronic nuclear DNA damage, could lead to NAD^+^ depletion and decrease sirtuin activity, likely contributing to age-associated pathophysiologies.

## It takes two to tango

**Figure 5:**
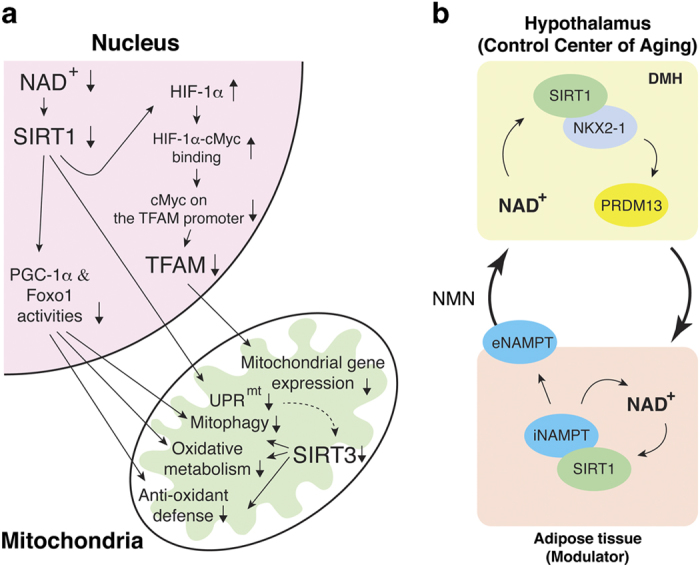
Importance of the communication between the nucleus and mitochondria at a cellular level (**a**) and between the hypothalamus and adipose tissue at a systemic level (**b**) in aging/longevity control. See details in text.

How does this intimate interplay between NAD^+^ and sirtuins contribute to aging/longevity control? At a cellular level, the communication between the nucleus and mitochondria seems to be compromised when NAD^+^ availability declines and thereby sirtuin activity decreases over age (Figure 5a). Interestingly, NAD^+^ deficiency causes a pseudohypoxic state through decreased SIRT1 activity.^57^ The defect in SIRT1 activity stabilizes HIF-1α, leading to an abnormal high level of HIF-1α. Because HIF-1α sequesters c-Myc, the gene encoding the mitochondrial transcription factor TFAM can no longer be activated by c-Myc (Figure 5a). In aged skeletal muscle, this defective TFAM expression causes significant reduction in mitochondrial gene expression, leading to mitochondrial metabolic dysfunction. Decreased SIRT1 activity also reduces the functions of PGC-1α and FOXO1, resulting in the reduction in mitochondrial biogenesis, oxidative metabolism, and anti-oxidant defense pathways.^74,75^ Furthermore, it has recently been demonstrated that SIRT1 and SIRT3 activities are involved in the mitochondrial unfolded protein response (UPR^mt^) pathway and mitophagy (Figure 5a).^25,76^ For instance, mammalian SIRT1, *C. elegans sir-2.1*, and NAD^+^ enhancement by NR all activate UPR^mt^ genes, such as *hsp-6* (*C. elegans*) and HSP60 (mammals), in worms, mammalian cells, and skeletal muscle stem cells.^25,77^ Interestingly, the activation of UPR^mt^ is tightly associated with mitonuclear protein imbalance, as defined by the decreased ratio between the nuclear DNA-encoded ATP5A and the mitochondrial DNA-encoded MTCO1 (cytochrome *c* oxidase subunit 1), and both UPR^mt^ and mitonuclear protein imbalance are proposed to have a critical role in aging and longevity control.^78^ SIRT3 appears to be a part of UPR^mt^, and mitochondrial proteotoxic stress increases SIRT3 protein levels, inducing mitophagy and anti-oxidant response.^76^ Therefore, the breakdown of the intimate interplay between NAD^+^ and sirtuins causes serious mitochondrial dysfunction, a hallmark of aging, through these nuclear-mitochondrial communication mechanisms.

At a systemic level, the communication between the control center of aging (the hypothalamus) and its modulator (adipose tissue) might be compromised when systemic NAD^+^ biosynthesis decreases (Figure 5b). As discussed in the previous section, adipose tissue remotely controls the hypothalamus through the secretion of eNAMPT. On the other hand, the hypothalamus also appears to control adiposity through a SIRT1-dependent signaling pathway. In the dorsomedial hypothalamus (DMH), SIRT1 and its binding partner Nkx2-1 have an important role in regulating aging and longevity.^27^ *PR domain containing 13 (Prdm13)* has recently been identified as one of the DMH-specific downstream target genes in the SIRT1/Nkx2-1 signaling pathway.^79^ Interestingly, DHM-specific *Prdm13*-knockdown mice exhibit decreased sleep quality and increased adiposity with no change in food intake, an interesting mimicry of age-associated pathophysiology. When NAD^+^ availability declines with age, it is likely that the secretion of enzymatically active eNAMPT would be affected in adipose tissue so that the hypothalamus would not be able to synthesize adequate levels of NAD^+^ from circulating NMN for its function (Figure 5b). This would lead to a decrease in *Prdm13* expression in the DMH, as observed indeed in aged hypothalamus,^79^ and an increase in adiposity. An interesting question is whether circulating eNAMPT levels would also increase as a compensatory response to sustain hypothalamic NAD^+^ levels. Although the study that tries to answer this question is currently underway, the systemic feedback loop between the hypothalamus and adipose tissue, mediated by the connection between NAD^+^ and SIRT1 in each tissue, is critical, contributing to the maintenance of physiological robustness. Therefore, the breakdown of this intimate interplay between NAD^+^ and sirtuins, particularly SIRT1, in each tissue likely leads to the breakdown of the inter-tissue communication between the hypothalamus and adipose tissue, resulting in systemic functional decline over age and eventually limiting lifespan in mammals.

## Concluding remarks

The tight functional connection between NAD^+^ and sirtuins has a critical role in regulating physiological robustness, and contributing to aging/longevity control in diverse organisms. Recent studies have demonstrated that NAD^+^ availability declines over age due to the defect in NAMPT-mediated NAD^+^ biosynthesis and the PARP-mediated NAD^+^ depletion, reducing sirtuin activities and affecting the communication between the nucleus and mitochondria at a cellular level and also the inter-tissue communication, particularly between the hypothalamus and adipose tissue, at a systemic level. These events likely cause age-associated pathophysiologies and contribute to the pathogenesis of diseases of aging. For this reason, supplementing key NAD^+^ intermediates, such as NMN and NR, is expected to mitigate age-associated functional decline and ameliorate a variety of age-associated pathophysiologies. The connection between NAD^+^ and sirtuins will provide deep mechanistic insight into how energy metabolism shapes the process of aging and determines lifespan in evolutionarily diverse organisms.
